# Powerful cell wall biomass degradation enzymatic system from saprotrophic *Aspergillus fumigatus*

**DOI:** 10.1016/j.tcsw.2024.100126

**Published:** 2024-05-21

**Authors:** Lige Tong, Yunaying Li, Xinke Lou, Bin Wang, Cheng Jin, Wenxia Fang

**Affiliations:** a National Key Laboratory of Non-food Biomass Energy Technology, Institute of Biological Sciences and Technology, Guangxi Academy of Sciences, Nanning, Guangxi, China; b College of Life Sciences, Hebei Innovation Center for Bioengineering and Biotechnology, Institute of Life Sciences and Green Development, Baoding, Hebei, China; c State Key Laboratory of Mycology, Institute of Microbiology, Chinese Academy of Sciences, Beijing, China

**Keywords:** *Aspergillus fumigatus*, Cell wall biomass, Degrading enzymes, Cellulose, Chitin

## Abstract

Cell wall biomass, Earth’s most abundant natural resource, holds significant potential for sustainable biofuel production. Composed of cellulose, hemicellulose, lignin, pectin, and other polymers, the plant cell wall provides essential structural support to diverse organisms in nature. In contrast, non-plant species like insects, crustaceans, and fungi rely on chitin as their primary structural polysaccharide. The saprophytic fungus *Aspergillus fumigatus* has been widely recognized for its adaptability to various environmental conditions. It achieves this by secreting different cell wall biomass degradation enzymes to obtain essential nutrients. This review compiles a comprehensive collection of cell wall degradation enzymes derived from *A. fumigatus*, including cellulases, hemicellulases, various chitin degradation enzymes, and other polymer degradation enzymes. Notably, these enzymes exhibit biochemical characteristics such as temperature tolerance or acid adaptability, indicating their potential applications across a spectrum of industries.

## Introduction

*Aspergillus fumigatus,* a common saprophytic fungus, plays a vital role in carbon and nitrogen recycling in the soil, serving as both a potent biological decomposer and an opportunistic pathogen causing invasive aspergillosis (Toyotome, 2019). Despite its pathogenic potential, studies highlight the remarkable ability of *A. fumigatus* to extract nutrients and energy from diverse sources, suggesting potential applications in various industries (Rhodes, 2006). While *A. fumigatus* cannot directly assimilate polysaccharides, it adopts a saprophytic lifestyle by secreting an array of cell wall-degrading enzymes, including cellulase, chitinase, phytase, chitosan enzyme, and more, with applications in enzyme engineering (Brugger et al., 2004, Saroj and Narasimhulu, 2022, Wang et al., 2023, Xia et al., 2001). These enzymes effectively break down cell wall biomass and polysaccharides into monomeric carbohydrates, facilitating absorption and metabolism (Nierman et al., 2005). Consequently, *A. fumigatus,* like other filamentous fungi, is utilized for the production of extracellular proteins (Adav et al., 2015).

*A. fumigatus* possesses a wealth of functional genes, exemplified by chitin synthase (Ries et al., 2017) and phytase (Mullaney et al., 2000), along with specific enzymes in its repertoire demonstrating remarkable thermal stability. This enzymatic diversity is advantageous for designing enzyme mixtures tailored for biomass utilization, including biofuel production, thereby establishing *A. fumigatus* as an exceptional source of industrial enzymes. However, unlike other industrially used *Aspergillus* species like *Aspergillus niger* and *Aspergillus oryzae*, certain strains of *A. fumigatus* are pathogenic, making them unsuitable for direct industrial enzyme production (Askew, 2008). Despite this limitation, recent research and reviews underscore the potential for studying biomass-degrading enzymes in *A. fumigatus* (Gonçalves et al., 2023). This review article aims to summarize the recent advancements in understanding these enzymes and their significant role in biomass degradation by *A. fumigatus*.

## Lignocellulose hydrolases

The growing cell wall in plants is characterized by its thinness, strength, and flexibility, with the extracellular layer playing a fundamental role in maintaining plant integrity. This structure comprises cellulose microfibrils ensconced in a hydrated matrix of intricate polysaccharides (cellulose-binding glycans) and a small proportion of structural proteins (Cosgrove, 2005). Lignocellulosic biomass, a primary constituent of plant cell walls, consists of cellulose, hemicellulose, and lignin (Hendriks and Zeeman, 2009, Singh et al., 2014, Van Dyk and Pletschke, 2012). Globally, projections indicate an annual production of over 20 billion tonnes of lignocellulosic biomass, establishing it as one of the most abundant and sustainable carbon resources on Earth (Liu et al., 2020). The predominant method for converting plant lignin biomass involves enzymatic hydrolysis technology. Effective hydrolysis of lignocellulosic biomass depends on the synergistic action of endoglucanases, exoglucanases and β-glucosidases (Kumar et al., 2008). While *Trichoderma reesei* is renowned for its production of endoglucanases, cellobiohydrolase, and β-glucosidases; however, its utility is hampered by low β-glucosidase activity, limiting further application. On the other hand, *A. niger* has the potential to produce cellulases with high β-glucosidase activity, yet its efficacy in cellulose hydrolysis is impeded by low endoglucanase expression (Liu et al., 2013).

Given the intricate composition and challenging breakdown of lignocellulosic biomass, employing multiple enzymes becomes imperative for effective deconstruction and utilization. *A. fumigatus* emerges as an important producer of lignocellulolytic enzymes, yielding substantial quantities of hydrolytic enzymes. The cellulase system, exemplified by the *A. fumigatus* Z5 cellulase system, boasts remarkable thermal stability and a broad pH range, offering distinct advantages. Notably, its enzymatic activity surpasses that of the highly cellulolytic strain *T. reesei* RUT30-C (Gouvêa et al., 2018).

### Cellulases

**Fig. 1:**
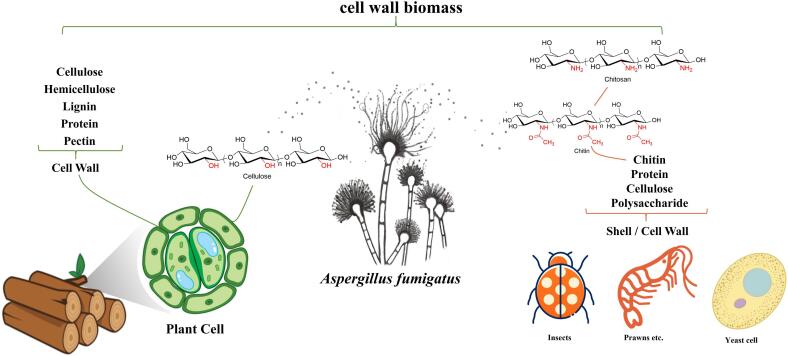
A schematic representation of the composition of various cell wall biomasses in nature.

Cellulose, a linear homopolymer of D-glucose linked by β-1,4 bonds (Fig. 1), stands out as a substrate with significant potential for conversion into biofuel and valuable chemicals (Tiwari et al., 2018). The enzymatic hydrolysis of cellulose relies on essential cellulases, which are categorized into three types: endoglucanase (E.C.3.2.1.4) (Manavalan et al., 2015), exoglucanase (E.C.3.2.1.91) (Srivastava et al., 2018), and β-glucosidase (E.C.3.2.1.21) (Rawat et al., 2014). Endoglucanase cleaves cellulose polymers' β-1,4-glycosidic bonds randomly, producing oligosaccharides. Exocellulase acts on both the reducing and non-reducing ends, generating smaller oligosaccharide units and cellobiose. The pivotal enzyme β-glucosidase plays a crucial role in cellulose hydrolysis by primarily cleaving cellobiose to release monosaccharide molecules (Singhania et al., 2013, Srivastava et al., 2018). Research indicates that cellulases from fungi, especially those from *A. fumigatus*, surpass their bacterial counterparts in terms of activity, heat resistance, and substrate heterogeneity (Budihal et al., 2016, Srivastava et al., 2018). *A. fumigatus* strains have been identified as capable producers of various cellulose hydrolases (Lin et al., 2017, Sharma Ghimire et al., 2016, Sharma et al., 2011). Notably, cellulases derived from selected *A. fumigatus* strain exhibit remarkable heat resistance further enhancing their potential in cellulose hydrolysis processes (Liu et al., 2011) (Table 1).

**Table 1:** The cellulose hydrolases from the reported *A. fumigatus* strains. NA, not available.

	Source	Temperature (℃)	pH	*K*_m_	Ref.
Endoglucanase					
Endoglucanase	Aspergillus fumigatus Fresenius	50	4.8	NA	(Parry et al., 1983)
*Af*-EGL7	*A. fumigatus Af*293	55	5.0	113.9 mg /mL	(Bernardi et al., 2018)
Eng2	*A. fumigatus*	24–40	5.0–5.6	0.8 mg/mL	(Hartl et al., 2011)
Egl2	*A. fumigatus*Z5	50	5.0	37.88 mg/mL	(Liu et al., 2011)
Egl3	*A. fumigatus* Z6	60	4.0	51.82 mg/mL	
Endoglucanase	*A. fumigatus* ABK9	50	5.0	6.7 mg/mL	(Das et al., 2014, Das et al., 2013)
Cel7	*A. fumigatus* DBiNU-1	60	5.0	NA	(Rungrattanakasin et al., 2018)
CMCase	*A. fumigatus* FBSPE-05	65	2.0	NA	(Grigorevski-Lima et al., 2009)
Endoglucanase	*A. fumigatus*	55	4.8	3.97 mM	(Mehboob et al., 2014)
CMCase	*A. fumigatus* JCM 10253	50	6.0	10.79 mg/mL	(Saroj et al., 2022)
CMCase	*A. fumigatus* A-16	70	6.0	NA	(ZHANG Kai-ping et al., 2022)
CMCase	*A. fumigatus* N2	65	4.0	NA	(Lin et al., 2017)
eng61	*A. fumigatus* MKU1	60	5.0	NA	(Meera et al., 2011)
Exoglucanase (cellobiohydrolase)					
Exoglucanase	*A. fumigatus*	55	5.5	4.34 mM	(Mahmood et al., 2013)
Exoglucanase	*A. fumigatus*	55	4.8	3.97 mM	(Mehboob et al., 2014)
*Af*Cel6A	*A. fumigatus*	50	5.0	0.9 ± 0.02 mg	(Dodda et al., 2020)
CBHI Cel7A	*A. fumigatus*	55	NA	NA	(Moroz et al., 2015)
β- Glucosidase					
cc	*A. fumigatus* KIBGE-IB33	65	7.0	3.39 ± 0.396 mg /mL	(Pervez et al., 2019)
nBgl3	*A. fumigatus* Z5	60	6.0	2.2 ± 0.26 mM	(Liu et al., 2012)
Exochitosanase	*A. fumigatus* IIT-004	40	5.5	8.0 mg/mL	(Sinha et al., 2016)
*exo*-beta-D-glucosaminidase	*A. fumigatus* S-26	50–60	3.0–6.0	1.0 mg/mL	(Jung et al., 2006)
β- Glucosidase	*A. fumigatus*	NA	4.0–5.0	0.84 mM	(Rudick and Elbein, 1975)
β-glucosidase	*A. fumigatus*	55	4.8	4.92 mM	(Mehboob et al., 2014)
β-glucosidase	*A. fumigatus* JCM 10253	60	5.0	32.56 mg/mL	(Saroj et al., 2022)
*Af*Bgl1.3	*A. fumigatus Af*293	40	6.0	7.6 ± 0.8 mM	(Pereira et al., 2023)

#### Endoglucanase and exoglucanase

Cultivated on lignocellulose, *A. fumigatus* demonstrates hightened cellulase activity (Parry et al., 1983, Sharma et al., 2011). In solid-state fermentation (SSF), this fungus efficiently employs untreated oil palm logs as its exclusive carbon source, leading to the production of cellulase enzymes, notably CMCase (carboxymethylcellulase; endoglucanase), FPase (filter paper activity; exoglucanase), and beta-glucosidase (Ang et al., 2015). The cellulase richness of *A. fumigatus* becomes apparent through quantitative secretome analysis, which identified 10 endoglucanases during the enzymatic degradation of cell wall cellulose in maize, wheat, and soybean (Sharma Ghimire et al., 2016). The whole genome sequence of *A. fumigatus* Af293 encompasses approximately 18 different genes encoding endoglucanases (Nierman et al., 2005). In contrast, the *A. fumigatus* Z5 strain, known for its robust cellulose degradation potential, reportedly harbors at least 25 endoglucanase genes. Notably, extracellular proteins from this strain contain 15 endoglucanases from families GH5, GH7, GH45, GH12, and AA9 (Miao et al., 2015b).

Despite the stability and efficiency of fungal-secreted endoglucanases at elevated temperatures, research on those secreted by *A. fumigatus* remains limited. However, *A. fumigatus* strain LMB-35Aa, isolated from the Peruvian Amazon rainforest, exhibits significant cellulase activity, including the presence of essential cellulose-degrading genes such as endoglucanase A, endoglucan protease B, and endoglucanase-like D (Paul et al., 2017). While the virulence and pathogenicity of *A. fumigatus* have traditionally hindered its application, the LMB-35Aa strain is recognized for its potent production of neutral/basic endoglucanase. The putrefactive status of this strain and its genetic differences from clinical isolates was further explored by Rebaza et al. Optimal conditions for endoglucanase production and activity of *A. fumigatus* LMB-35Aa were found to be 37 °C and pH 7.6. Notably, this temperature inhibited virulence factors such as *gliZ*, *atrF* and *medA,* with only minor expression of virulence-related genes observed.

Endoglucanases from *A. fumigatus* typically exhibit high optimal reaction temperatures ranging from 45 to 60°C. Das *et al*. purified a halotolerant endoglucanase with a calculated molecular weight of approximately 56.3 kDa from a culture extract of *A. fumigatus* ABK9. This enzyme showed maximum activity at 50°C and pH 5.0, demonstrating high stability within a pH range of 4.0–7.0 and up to a NaCl concentration of 3.0 M (Das et al., 2013). In another study, Aline Vianna Bernardi *et al*. heterologously expressed the gene encoding a GH7 family endoglucanase (Afu6g01800) from *A. fumigatus* Af293 in *Escherichia coli*. The enzyme exhibited a broad optimal temperature range spanning from 40 to 60°C (Bernardi et al., 2018). Additionally, when the endoglucanase gene from the heat-resistant *A. fumigatus* DBiNU-1 strain was expressed in the lactic acid-producing *Kluyveromyces lactis*, the purified recombinant endoglucanase displayed peak activity at 60°C (Rungrattanakasin et al., 2018).

Cellulase enzymes originating from *A. fumigatus* exhibit remarkable temperature tolerance. Notably, an endoglucanase belonging to the AA9 family (formerly GH61), isolated from alkali-resistant *A. fumigatus* MKU1 (derived from pulp and paper industry waste), demonstrating resilience at temperatures up to 70°C (Meera et al., 2011). Another *A. fumigatus* strain A-16, produces enzymes with an optimum reaction temperature of 70°C, retaining over 80% activity even after heat treatment at 80°C for 90 min (ZHANG Kai-ping et al., 2022). The endoglucanase from this strain displays optimal activity between 55 and 80°C. This property of high-temperature tolerance is advantageous for constructing cost-effective and stable strains suitable for industrial conditions, making them promising candidates for various industrial applications (Bhiri et al., 2010, Maheshwari et al., 2000, Rungrattanakasin et al., 2018).

While the current research landscape on cellulosic disaccharide hydrolases has predominantly focused on genera *Aspergillus, Trichoderma, and Penicillium* (Bhiri et al., 2010, Ogata et al., 2010, Wang and Xia, 2011), there exists a relative scarcity of studies on exoglucanases from *A. fumigatus*. Nevertheless, several successfully expressed cellobiose hydrolases from *A. fumigatus,* including *Af*Cel7A (Moroz et al., 2015), *Af*Cel6A (Dodda et al., 2020), CBH from *A. fumigatus* NITDGPKA3 (Dodda et al., 2016), and *exo*-β-glucanase produced during the solid-state fermentation of wheatgrass, have been identified (Mahmood et al., 2013). Their optimal reaction temperatures cluster around 50-55°C, aligning with the broader trend of higher reaction temperatures observed in endoglucanases.

Cellobiose hydrolase, a cellulose disaccharide hydrolase, targets the crystalline region of cellulose, playing a pivotal role in the hydrolysis of natural cellulose into cellulose disaccharides. The industry has perennially grappled with challenges related to pH stability, thermal stability, and low conversion efficiency. The crystal structure of *A. fumigatus* CBHI Cel7A has undergone analysis, revealing a classic β sandwich structure in its three-dimensional configuration. *Af*Cel7A demonstrates commendable stability, retaining high activity even after incubation at 65°C (Moroz et al., 2015). In addition, Dodda *et al.* successfully achieved a comprehensive enhancement in the catalytic activity and stability of the mutant N449V through protein engineering (Dodda et al., 2016). These advancements not only contribute to overcome industry challenges but also establish a robust foundation for ongoing research and the practical application of cellobiose hydrolase.

#### β-glucosidases

In the early 1970s and 1975, Parry *et al*. successfully characterized two sources of β-glucosidase from *A. fumigatus* (Rudick and Elbein, 1975, Rudick and Elbein, 1973)*.* The β-glucosidase derived from *A. fumigatus* employs an acid-base catalytic mechanism, facilitating the hydrolysis of β-glucosidic bonds and releasing glucose from the substrate. Structurally, it typically consists of an N-terminal signaling peptide, a catalytic domain, and a C-terminal domain, with a molecular weight falling within the range of 60–80 kDa (Agirre et al., 2016).

A heat-stable natural β-glucosidase (nBgl3) was isolated from crude extracts of *A. fumigatus* strain Z5, showing optimal activity at pH 6.0 and 60°C, along with stability at pH 4–7 and temperature ranging from 50 to 70°C (Liu et al., 2012). Additionally, the genome sequence of *A. fumigatus* LMB-35Aa also revealed the presence of β-glucosidase (Paul et al., 2017). Fungal cellulase activity typically peaks between 50 and 60°C, stabilizing at 50 to 55°C (Monteiro et al., 2020). *A. fumigatus* β-glucosidase follows a similar pattern, with a maximum temperature range usually between 40 and 60°C (Saroj et al., 2022) (Table 1).

*A. fumigatus* Z5 emerges as an excellent candidate for cellulase production (Liu et al., 2013, Liu et al., 2012). Both nBgl3 and rBgl3, its heat-resistant β-glucosidases, retain more than 40% and 50% of their activity, respectively, even when treated at 70°C for one hour. The degradation of cellulose to produce glucose typically necessitates the coordinated action of at least three distinct and complementary cellulases: endoglucanase, exoglucanase and β-glucosidase. Among these, β-glucosidase plays a crucial role in preventing the accumulation of cellobiose and alleviating its inhibitory effect on cellulase activity. This enzyme ultimately determines the efficiency of the cellulose saccharification process, and serves as a key catalyst for promoting cellulose degradation. Therefore, the stability of β-glucosidases is essential for ensuring the robustness of industrial bioenergy utilization process. These β-glucosidases (nBgl3 and rBgl3) hold promise for diverse applications in bioenergy and food processing, consequently.

### Hemicellulase

Hemicellulase, a crucial player in the natural degradation of cell wall biomass and lignocellulosic materials by fungi, primarily targets hemicellulose, which ranks as the second most abundant polysaccharide after cellulose. This complex structure comprises various components such as xylan, glucan, galactose-glucan, and arabinogalactan (Pauly et al., 2013). Xylan, a significant polymer within hemicellulose, requires a complexed enzymatic system (Gupta et al., 2016), including β-1,4-endoxylanase, β-xylosidase, acetyl xylan esterase, arabinose, α-glucuronidases, ferulic acid esterase, and p-coumaric acid esterase. These enzymes collaborate synergistically to break down xylan into its constituent sugars (Bagewadi et al., 2016). The Carbohydrate-Active Enzyme (CAZy) database categorizes xylanases into glycoside hydrolase (GH) families 5, 7, 8, 10, 11, 26, 30, and 43. Notably, the majority of xylanases fall within GH families 10 and 11 (Lagaert et al., 2014).

Backbone-hydrolyzing enzymes, namely *endo*-β-1,4-xylanase and β-D-xylosidase, play a vital role in catalyzing the hydrolysis of β-1,4-linked d-xylopyranose units from the homopolymeric backbone of xylan. Complementing these activities are branch-degrading enzymes, including α-L-arabinofuranosidase, α-D-glucuronidase (Rosa et al., 2013), acetylxylan esterase, and phenolic acid esterases (such as feruloyl or coumaroyl esterases) (Malgas et al., 2021). The central enzyme in this process, *endo*-β-1,4-xylanase, is primarily classified into the glycoside hydrolase families GH10 and GH11. GH10 features a (β/α)8-TIM barrel structure, while GH11 exhibits a β-jelly structure (Lombard et al., 2014).

Xylanases exhibit significant diversity, with various microorganisms capable of simultaneously producing different types of xylanases. Fungi, including *A. fumigatus*, are known to synthesize and secrete xylanases into the external environment (Damasio et al., 2017, Damis et al., 2019; Miao et al., 2015b). In a study by Miao et al., 10 different hemicellulases from *A. fumigatus* Z5 were identified and quantified. Among them, four *endo*-xylanases, two xyloglucosidases, one acetyl-xylan esterase, and one α-L-arabinofuranosidase were identified as crucial participants in xylan degradation (Miao et al., 2015b). Xylanases from *A. fumigatus*, like other enzymes, exhibit high reaction stability and a broad pH range.

In industrial applications, finding fungal xylanases that are both alkaline and heat-stable proves challenging. Typically, most bacterial and fungal GH11 xylanases are alkaline, while GH10 xylanases are acidic. Through a computational analysis of the *A. fumigatus* genome (KEY83365), Dodda et al. identified alkaline xylanases belonging to the GH10 family. The study highlighted that, GH11 xylanase showed higher binding energy than GH10 xylanase. However, with a few exceptions, GH10 xylanases demonstrate a higher number of hydrogen bonds, salt bridges, and helices compared to GH11 counterparts, contributing to their superior heat resistance (Dodda et al., 2021). Furthermore, GH10 xylanases typically possess a higher molecular weight than GH11 xylanases. A thermotolerant albino strain of *Aspergillus* (*A. fumigatus* var. niveus), isolated from Brazilian rainforest composted floors, yielded a GH10 xylanase (AFUMN-GH10) that was cloned and heterologously expressed. AFUMN-GH10 exhibits an optimal temperature of 60 °C and retains over 60% of its maximum activity within the temperature range of 30 to 75°C. Xylanases belonging to the GH10 family from other filamentous fungi also showed optimal reaction temperatures ranging from 60 to 75°C (Velasco et al., 2019). However, this strain has the same pathogenicity as *A. fumigatus* Af293 (Couger et al., 2018). Therefore, careful selection of a heterologous host for protein expression is imperative.

*fumigatus* not only displays xylanase activity but also features other hemicellulases, including galactosidase. Galactosidase, a member of the glycoside hydrolase (GH) family, plays a role in catalyzing the hydrolysis of galactosides into monosaccharides. An α-galactosidase purified from the thermotolerant *A. fumigatus* strain IMI 385708 was identified to catalyze the glycosylation of internal sugar residues in oligosaccharides (Puchart and Biely, 2005). Utilizing recombinant technology, the genes encoding two α-L-arabinofuranosidases (*AbfI* and *AbfII*) from GH family 62, as identified in the *A. fumigatus* genome, were expressed in *Pichia pastoris*. Following purification and characterization, both ABFI and ABFII displayed optimum temperatures of 37°C and 42°C, respectively, with an optimal pH range of 4.5 to 5.0. Notably, ABFII demonstrated higher thermostability (Pérez and Eyzaguirre, 2016). These findings underscore the diverse enzymatic capabilities of *A. fumigatus* in the context of hemicellulose degradation (Table 2) and offer valuable insights for industrial applications.

**Table 2:** Reported hemicellulose hydrolases from *A. fumigatus* strains. NA, not available.

Type	Name	Source	GH Family	Temperature (℃)	pH	Ref.
Xylanase	*Af*xynG1	*A. fumigatus* RT-1	11	50	5	(Damis et al., 2019)
	*Af*XylA10	*A. fumigatus* JL16	10	53	7	(Wang et al., 2021)
	xylanase	A. fumigatus SBC4	NA	60	4	(Santos et al., 2015)
	xylanase	*A. fumigatus* Z5	NA	60	3–6	(Miao et al., 2015a)
	xylanase	*A. fumigatus* R1	NA	50	5	(Deshmukh et al., 2016)
	xylanase	*A. fumigatus* MA28	NA	50	8	(Bajaj and Abbass, 2011)
	Xyn10A	*A. fumigatus* Z5	10	90	6	(Miao et al., 2018)
	AFUMN-GH10	*A. fumigatus* var. niveus	10	60	5	(Velasco et al., 2019)
	xynf11	*A. fumigatus* MKU1	11	60	6	(Jeya et al., 2009)
	*Af*-XYNA	A. fumigatus *Af*293	10	75	5	(Chang et al., 2017)
	XynAF1	*A. fumigatus* Z5	10	90	5	(Li et al., 2021)
	xylanase	*A. fumigatus* N2	NA	65	6	(Lin et al., 2017)
	α-Galactosidase	*A. fumigatus* IMI 385708	27	50	4.5	(Puchart and Biely, 2005)
α-L- Galactosidase arabinofuranosidase	ABFⅠ	*A. fumigatus* wmo	62	37	4.5–5	(Pérez and Eyzaguirre, 2016)
	ABFⅡ	*A. fumigatus* wmo	62	42	4.5–5	

## Chitin hydrolases

Chitin, constituting 10–30% of fungal cell walls, stands as the principal component in these structures (van Leeuwe et al., 2020). This linear polymer consists of β-(1,4)-linked N-acetylglucosamine (Fig. 1), and chitin molecules are classified into three types based on the arrangement of adjacent sugar chains: α-Chitin, β-Chitin, and γ-Chitin (Chen et al., 2023). Although chitin is insoluble in water and challenging to decompose, its deacetylated product, chitosan, features numerous free amino groups with positively charged molecules, rendering it soluble in acidic and neutral aqueous solutions (Elango et al., 2023). Despite minimal difference in molecular structure, both chitin and chitosan play crucial roles in providing structural stability for fungal cell walls (Brown et al., 2020). Within the realm of chitin metabolism, chitinase, chitin deacetylase, and chitosanase are essential members for chitin hydrolases. Currently, there is limited research on chitin hydrolases derived from *A. fumigatus* compared to other filamentous fungi (Table 3). This observation underscores the potential for further exploration and understanding of *A. fumigatus* chitinolytic enzymes and their implications in fungal cell wall dynamics.

**Table 3:** Reported chitin hydrolases from *A. fumigatus* strains. NA, not available.

Name	Source	GH/ CE Family	Temperature (℃）	Substrate	Activity	Ref.
Chitinase						
chitinase	*A. fumigatus* YJ-407	GH 18	60	Powder Chitin	NA	(Xia et al., 2001)
				Colloidal Chitin	3.36 U/mg	
Δ30*Af*ChiJ	*A. fumigatus* df673	GH 18	45	Powder Chitin	3.82 ± 2.91 mU/ml	(He et al., 2022)
				Colloidal Chitin	110.47 ± 0.07 mU/ml	
*Af*Chi28	*A. fumigatus* df347	GH 18	45	Powder Chitin	0.0137 mU/mg	(Wu et al., 2022)
				Colloidal Chitin	52.414 mU/mg	
*Af*chiB1	*A. fumigatus* YJ-407	GH 18	60	Powder Chitin	0.98 U/mg	(Lü et al., 2009)
				Colloidal Chitin	11.01 U/mg	
chi44	*A. fumigatus* YJ-407	GH 18	60	Powder Chitin	NA	(Ying, 2004)
				Colloidal Chitin	5.64 U/mg	
Chitosanase						
CSN-P	A. fumigatus Y2K	GH 75	55–65	Chitosan	25,000 U/ml	(Chen et al., 2012)
chitosanase I	*A. fumigatus* KB-1	NA	60	NA	NA	(Eom and Lee, 2003)
chitosanase Ⅱ	*A. fumigatus* KB-2	NA	70	NA	NA	
chitosanase Ⅱ	A. fumigatus ATCC13073	GH 75	40	Chitosan	8.8 U/mg	(Hirano et al., 2012a)
csnw2	Aspergillus sp. W-2 (CGMCC7018)	GH 75	55	NA	NA	(Zhang et al., 2015)
chitosanase I	*A. fumigatus* KH-94	NA	70–80	Chitosan	152 U/mg	(Kim, 1998)
chitosanase Ⅱ		NA	50–60	Chitosan	297 U/mg	
Chitin deacetylases						
Cod4	*A. fumigatus* YJ-407	CE 4	37	GlcNAc4	18.98 U/mg	(Xie et al., 2020)
Cod7			NA	NA	NA	

### Chitinase

Chitinases (EC 3.3.1.14) are widespread in viruses, fungi, and bacteria, with each source exhibiting distinct functions. Those produced by filamentous fungi are commonly associated with processes such as cell wall cleavage, spore formation and germination, mycelial growth, and fungal parasitism (Flach et al., 1992). Given their pivotal physiological roles and broad application potential, chitinases have become a highly researched topic in recent years (Di Giambattista et al., 2001). As the major structural component within the cell walls of all human pathogenic fungi, chitin polymers are absent in mammals. This absence positions chitin-metabolizing enzymes as promising targets for the development of novel antifungal agents. Chitinases are classified into two categories based on different hydrolysis modes: endogenous chitinases (EC 3.2.1.14) and exogenous chitinases (EC 3.2.1.29) (He et al., 2022). Further differentiation is observed in protein sequence, structure, and catalytic mechanism, placing chitinases into two families, GH18 and GH19 (Henrissat, 1991), with fungal chitinases predominantly belonging to GH18 (Khan et al., 2015). Among the GH18 chitinases identified in microorganisms, varying degrees of glycosylation activity have been reported (Aguilera et al., 2003). The versatile functions of chitinases and their potential as targets for antifungal therapies underscore the importance of understanding their mechanisms and activities, especially within the context of fungal pathogenesis.

The genome of the fungus *A. fumigatus* is known to harbor a rich array of chitinase genes, numbering at least 17 (He et al., 2022). A comprehensive study identified a complex chitinase catabolism in *A. fumigatus*, revealing 14 distinct chitinases through phylogenetic analysis (Taib et al., 2005). The successful cloning and overexpression of the 45 kDa chitinase gene ChiB1 from *A. fumigatus* in *Saccharomyces cerevisiae*, revealed a retaining mechanism typical of GH18 family chitinases (Jaques et al., 2003). Additionally, a heat-resistant chitinase isolated from *A. fumigatus* YJ-407 displayed stability within a pH range of 4.0 to 8.0, with peak activity at pH 5.0 and an optimal temperature of 60°C. This enzyme exhibited *endo*-chitinase, *exo*-chitinase, and transglycosylation activities (Xia et al., 2001). Unlike most bacteria, fungal chitinases typically exhibit optimal activity under acidic conditions.

While limited studies on chitinases in marine fungi have hindered the understanding of their potential, recent investigations successfully identified and characterized chitinase genes in two marine *A. fumigatus* strains, df673 (He et al., 2022) and df347 (Wu et al., 2022). *Af*ChiJ and *Af*Chi28, derived from these marine strains, stand out as acid-, salt-, and temperature-tolerant bifunctional enzymes with endo- and *exo*-endonucleation capabilities. *Af*ChiJ, with an optimum temperature of 45°C in colloidal chitin and a pH of 4.0, maintained high activity (≥97.96%) in 1–7% NaCl. These findings suggest that marine fungal chitinases hold significant potential for the high-value utilization of chitin biomass and may emerge as promising candidates for applications in green industries. The exploration of marine fungal chitinases expands our understanding of their diverse characteristics and applications, potentially unlocking valuable opportunities in sustainable and environmentally friendly industrial processes.

### Chitosanase

Chitosan, a derivative of chitin through deacetylation, is characterized by its composition of β-(1→4)-linked D-glucosamine and N-acetyl-D-glucosamine units (Zhang et al., 2019) (Fig. 1). This biopolymer plays a crucial structural role in fungal cell walls, potentially influencing the virulence of pathogenic fungi (Beck et al., 2014). Chitosanase (EC 3.2.1.132) targets the β-1,4-glycosidic bond in chitosan, releasing oligosaccharides with heightened biological activity (Cheng et al., 2006). Unlike bacterial chitosanases, the physiological function of fungal chitosanases remains unclear, primarily because fungi expressing chitosanase are unable to grow on chitosan (Sugita et al., 2012). However, Chitosan serves as a pivotal mediator and target in fungal pathogenesis, making it an attractive therapeutic target for fungicide development.

In the CAZy database, chitosanases are categorized into GH3, GH5, GH7, GH8, GH46, GH75, and GH80 families based on amino acid sequence homologies (Xu et al., 2023). The GH75 family predominantly includes *A. fumigatus*-derived chitosanases, with the endonuclease CSN being the first chitosanase studied in this family (Cheng et al., 2006). However, CSN is predominantly expressed as inclusion bodies in *E. coli* BL21 (DE3), limiting downstream purification and industrial applications. To overcome this challenge, Chen et al. explored *Pichia pastoris* as a host for CSN secretion expression. This approach yielded high activity, with 3 g of the enzyme converting 60 kg of chitosan into oligosaccharides within 24 h at 200°C. Remarkably, the enzyme exhibited a half-life of 32 min at 100°C. This research highlights the potential of fungal chitosanases for industrial applications and provides insights into their production and stability under extreme conditions.

Chitosanases display diverse specificities in the hydrolysis of β-glycosidic linkages within partially N-acetylated chitosan molecules, leading to the classification into chitosanase I (cleaving GlcN-GlcN and GlcNAc-GlcN bonds), chitosanase II (cleaving only GlcN-GlcN bonds), and chitosanase III (cleaving GlcN-GlcN and GlcN-GlcNAc bonds) (Hirano et al., 2012b). *A. fumigatus* strains, including KH-94 (Kim, 1998), Y2K (Cheng and Li, 2000) and KB-1 (Eom and Lee, 2003) express chitosanases with heightened hydrolytic activity towards nearly 100% deacetylated chitosan compared to partially deacetylated forms. In KB-1 (Eom and Lee, 2003) and KH-94 (Kim, 1998), two distinct chitosanase types, chitosanase I and chitosanase II, have been identified. These enzymes significantly differ in molecular weight, optimal pH temperature, enzymatic properties, and reaction products. Furthermore, chitosanase II with a molecular mass of 23.5 kDa was purified from the culture filtrate of *A. fumigatus* ATCC13073, showing an optimum pH and temperature of 6.0 and 40°C, respectively. This enzyme shares an N-terminal amino acid sequence identical to other GH 75 family *Aspergillus* chitosanases (Hirano et al., 2012b).

Chitosanases GH75 from *Aspergillus* sp. exhibit a notable ability to hydrolyze chitosan into DP 2–7 chitooligosaccharides, showcasing potential applications in the field of medicine (Zhang et al., 2015). Notably, the promotion of chitosanase production was observed by analyzing the phenotype of an *A. fumigatus* strain with a deleted *CsnB* gene. Recombinant protein experiments further unveiled the presence of an IgG response protein, suggesting that *A. fumigatus* chitosanase CsnB may have utility in serological diagnosis (Beck et al., 2014). This information underscores the diverse applications and potential medical relevance of chitosanases produced by *Aspergillus* species, particularly *A. fumigatus*.

### Chitin deacetylase

Chitin and chitosan differ primarily in their degree of acetylation, with polymers having a degree at or above 50% commonly known as chitin, and those with a degree below 50% referred to as chitosan (Kasaai, 2009). Chitin deacetylase (CDA), a key enzyme responsible for converting chitin to chitosan, belongs to the carbohydrate esterase family 4 (CE4) (EC 3.5.1.41) (Caufrier et al., 2003). Within fungal genomes containing chitin, the CE4 family is prevalent, and CDA plays a crucial role in cell wall morphogenesis and spore formation (Baker et al., 2007). Phytopathogenic fungi are able to evade host immune defenses during infection by secreting CDA, thus CDA is essential for fungal virulence (Liu et al., 2023).

In the genome of *A. fumigatus*, seven putative CDA exist: CDA1 (AFUA_1G15280), CDA2 (AFUA_6G05030), CDA3 (AFUA_3G07210), CDA4 (AFUA_5G11410), CDA5 (AFUA_4G09940), CDA6 (AFUA_6G10430), and CDA7 (AFUA_5G09130). Among them, CDA1, CDA2, and CDA7 are predicted to be intracellular proteins, while CDA3, CDA4, and CDA6 have predicted N-terminal peptide signals, suggesting potential secretion into the extracellular domain (Mouyna et al., 2020). Xie et al. cloned and expressed two of these genes, *Cod4* and *Cod7*, in *E. coli* (Xie et al., 2020). The study revealed that *A. fumigatus* chitin deacetylase Cod4 selectively deacetylates (GlcNAc)4, while Cod7 is inactive. The absence of Cod4, Cod7, or both resulted in abnormal polarity and increased conidia, suggesting that CDA is likely associated with the conidiation of *A. fumigatus*. However, research has shown that these CDA genes are not essential for the virulence of *A. fumigatus* (Xie et al., 2020). In contrast, the *Cryptococcus neoformans* chitin deacetylase Cda1 is crucial for virulence (Upadhya et al., 2018).

## Other exocrine proteins

The structural foundation of fungal walls is layered, featuring a robust core scaffold comprised of fibrous and gelatinous carbohydrate polymers. This scaffold serves as the base to which various proteins and additional surface components are added, forming branched chains that attach to proteins and other polysaccharides. The specific composition of these components varies among fungal species (Gow et al., 2017). In the case of *C. albicans* and *S. cerevisiae*, β-(1,6) glucan serves as a linker molecule, binding cell wall proteins (CWPs) to the β-(1,3) glucan-chitin backbone through glycosylphosphatidylinositol residues. In yeast cells, CWPs constitute 30––50% of the cell wall’s dry matter (Klis et al., 2001).

### Protease

Proteases, enzymes essential for peptide bond hydrolysis, are highly valued in industry for their remarkable activity, specificity, stability, and tolerance to diverse conditions such as temperature, metal ions, surfactants, and organic solvents. These proteases are categorized into various groups, including cysteine, serine proteases, metallo-, and aspartic acid proteases, among others (Morya et al., 2012). The filamentous fungus *A. fumigatus* is renowned for secreting numerous allergens with protease activity, and this secretion is associated with various allergic conditions (Farnell et al., 2012). Recent research highlights that the quantity, type, and activity of the main allergen proteases secreted by *A. fumigatus* are influenced by physiological substrates or specific protein substrate reactions. *A. fumigatus* demonstrates the ability to sense different protein substrates in its environment, regulating the secretion of proteases accordingly. For example, in a culture medium where collagen serves as the sole nitrogen and carbon source, *A. fumigatus* predominantly secretes serine alkaline protease (ALP) and metalloproteinase (MEP) (Jaton‐Ogay et al., 1994). Additionally, beyond complex protein substrates, the fungus adjusts its protease secretion based on the pH of the growth medium (Sriranganadane et al., 2010). At neutral pH values, *A. fumigatus* secretes a set of proteases, including neutral and alkaline endonucleases, while acidic pH values promote the secretion of aspartate protease. This dynamic regulation of protease secretion in response to environmental cues highlights the adaptability of *A. fumigatus* and its ability to tailor its enzymatic arsenal to the prevailing conditions.

*A. fumigatus*, a highly pathogenic fungus, is well-known for causing invasive aspergillosis (IA), aspergilloma, and allergic bronchopulmonary aspergillosis (ABPA). There exists a demonstrated correlation in mice between the elastase produced by *A. fumigatus* strains and their capacity to cause IA. This correlation strongly suggests that the toxicity of *A. fumigatus* is closely linked to the production of extracellular proteases (Monod et al., 1991). Notably, the secretion of these proteases is considered essential for overcoming the host protein barrier (Gifford et al., 2002). Compared to other fungi within the *Aspergillus* genus, such as *A. oryzae*, *A. niger*, *A. flavus*, and *A. tereus*, *A. fumigatus* stands out with an increasing number of identified proteases in its secretome. These identified proteases exhibit the capability to hydrolyze various protein substrates, including the degradation of smaller peptide segments. This characteristic is believed to be associated with *A. fumigatus*'s efficient acquisition of nitrogen sources (Reeves et al., 2004). In summary, investigating the pathways related to *A. fumigatus* proteases or protease expression holds promise as a potential target for future drug therapy in fungal infections. The understanding of *A. fumigatus*'s proteolytic mechanisms may offer valuable insights into developing therapeutic strategies to combat fungal infections.

### Phytase

Phytic acid, a phosphorylated derivative of myo-inositol, plays a crucial role as the primary storage form of phosphorus in plant seeds. Myo-inositol phosphates, which include phytic acid, exhibit a diverse array of functions in plants, ranging from acting as signaling molecules and osmoprotectants to being integral components of cell walls (Hegeman et al., 2001). In most plants, the primary storage form of phosphorus is phytate, specifically myo-inositol hexakisphosphate. However, monogastric animals, such as pigs and birds, encounter challenges in directly utilizing phytate due to insufficient levels of digestive enzymes in the intestine (Tahir et al., 2012). To address the phosphorus requirements for the growth of these animals, additional inorganic phosphorus may be added, but this can lead to significant ecological problems. Phytase (EC 3.1.3.8), belonging to the histidine phosphatase family, is primarily found in microorganisms and plants. These enzymes catalyze the release of phosphate from phytic acid, the main form of phosphorus storage in plants. Consequently, the supplementation of feed with phytase can enhance the utilization rate of phytate in monogastric animals while simultaneously reducing phytate phosphorus pollution (Han et al., 2018).

In the realm of phytase, possessing robust acid stability and thermal stability is imperative (Tomschy et al., 2002). While *A. niger* phyA phytase (AnP) demonstrates superior enzyme activity (102.5 U/mg) compared to *A. fumigatus* ATCC 13073*A* wild-type phytase (Afp), Afp stands out for its remarkable thermal stability and a broad range of optimal pH values (Gu et al., 2007). Even when exposure to 100°C for 20 min, Afp retains 90% of its initial enzyme activity (Pasamontes et al., 1997). Notably, Afp demonstrates the ability to hydrolyze various substrates such as phytate, fructose, glucose 6-phosphate, etc.(Tomschy et al., 2002). In a comprehensive structural analysis of Afp, it consists of a small α-structure domain and a large α/β-structure domain composition, sharing a similar folding pattern to AnP. Liu et al. identified a larger catalytic pocket of Afp compared to *E. coli* phytase, potentially explaining its broader substrate specificity (Liu et al., 2004). Additionally, an acid phytase has been cloned from *A. fumigatus* ATCC34625 (Rodriguez et al., 2000), *A. fumigatus* NF191 (Gangoliya et al., 2015) and *A. fumigatus* WY-2 (Wang et al., 2007). This discovery holds significant implications for the application of phytase in the feed industry, showcasing the potential for enhanced enzyme performance and versatility in various feed formulations.

## Conclusion and outlook

As a widely distributed saprophytic fungus in nature, *A. fumigatus* faces the challenge of limited directly utilizable monosaccharides in its environment. In response to this, it employs a strategy of secreting substantial amounts of proteins to break down organisms in its habitat, including cell wall biomass, acquiring nutrients for growth. While other filamentous fungi like *T. reesei*, *Penicillium oxalate*, and *A. niger* also secrete hydrolytic proteins, *A. fumigatus* has sparked industrial interest due to its remarkable enzyme production performance and the heat and acid resistance properties of its proteins. Enzymes produced by *A. fumigatus* exhibit thermal stability across diverse environments, including high-temperature compost piles, laboratory settings, and preclinical conditions (Bernardi et al., 2018, Kadam and Drew, 1986; Miao et al., 2015b). This characteristic may be attributed to the fungus's robust environmental resilience and adaptability. While pathogenicity poses a barrier to direct utilization in production, the versatile and efficient enzyme system of *A. fumigatus* can be tailored to develop optimized enzyme systems for industrial carbohydrate degradation. Moreover, the use of safe fungal heterologous expression systems, such as *Pichia pastoris*, offers a solution to this challenge.

Beyond producing large-molecule enzymes, *A. fumigatus* can generate peptides with antifungal activity, prompting further research and development. Efforts to overcome *A. fumigatus*'s pathogenicity focuse on developing a safe degradation system for industrial production. This involves screening non-pathogenic high-yield hydrolytic enzyme strains and mitigating their toxicity through genetic engineering or efficient heterologous expression. By implementing thorough detection and pre-treatment protection measures, the untapped potential of *A. fumigatus* in enzyme engineering can be harnessed across research, development, production, and application of its enzymes. This approach aims to leverage the beneficial aspects of *A. fumigatus*'s enzyme system while addressing safety concerns for industrial applications, particularly in cell wall and cell surface modification.

## Funding

This work was supported by Guangxi Science and Technology Base and Talent Special Project (AD23026267) to L.T, and Guangxi Natural Science Foundation (2023GXNSFFA026011, 2020GXNSFDA238008) to W.F.

## CRediT authorship contribution statement

**Lige Tong:** Writing – review & editing, Writing – original draft, Visualization, Investigation, Funding acquisition, Formal analysis, Data curation, Conceptualization. **Yunaying Li:** . **Xinke Lou:** . **Bin Wang:** Supervision, Resources, Formal analysis, Conceptualization. **Cheng Jin:** Writing – review & editing, Investigation, Formal analysis. **Wenxia Fang:** Writing – review & editing, Supervision, Resources, Funding acquisition, Conceptualization.

## Declaration of competing interest

The authors declare that they have no known competing financial interests or personal relationships that could have appeared to influence the work reported in this paper.
